# Single-Stage Endovascular Treatment of Severe Cranial Artery Stenosis Coexisted With Ipsilateral Distal Tandem Intracranial Aneurysm

**DOI:** 10.3389/fneur.2022.865540

**Published:** 2022-05-18

**Authors:** Hongyang Ni, Zhihong Zhong, Jun Zhu, Hong Jiang, Jinqing Hu, Dong Lin, Liuguan Bian

**Affiliations:** Department of Neurosurgery, Ruijin Hospital, Shanghai Jiao Tong University School of Medicine, Shanghai, China

**Keywords:** cranial artery stenosis, intracranial aneurysm, coexistence, single-stage, endovascular treatment

## Abstract

**Objective:**

The coexistence of severe cranial artery stenosis and ipsilateral distal tandem intracranial aneurysm is an unusual phenomenon. Currently, there is no consensus to provide treatment guidelines for concomitant lesions. This study aims to evaluate the safety and effectiveness of single-stage endovascular treatment in patients under this special condition.

**Methods:**

We illustrated a case series of 10 patients with the coexistence of severe cranial artery stenosis and ipsilateral distal tandem intracranial aneurysm in our hospital. And a systematic PubMed search of English-language literature published between 1990 and 2021 was carried out using the keywords: “(carotid OR vertebral OR subclavian artery stenosis) AND (aneurysm) AND (coincident OR coexist OR concomitant OR simultaneous OR ipsilateral).” Clinical information, including age, gender of the patients, as well as symptoms (artery stenosis or aneurysm), localization of artery stenosis and aneurysm, treatment, and outcome, were collected and analyzed.

**Results:**

In the majority of the patients, symptoms were attributed to severe artery stenosis, and the coexisted lesions were located in the anterior circulation system. Most patients achieved an excellent clinical outcome, and no death was observed. No differences were found in a prognosis between single-stage or multiple-stage endovascular treatment.

**Conclusions:**

A single-stage endovascular procedure is technically feasible and effective to treat the coexistence of severe cranial artery stenosis and ipsilateral distal tandem intracranial aneurysm in the anterior circulation as well as in the posterior circulation.

## Introduction

The coexistence of severe cranial artery stenosis and ipsilateral distal tandem intracranial aneurysm is an unusual phenomenon and is usually detected incidentally. When occurs, it leads to a quandary regarding both the clinical management and the surgical approach. Although various treatment options have been proposed, there is still no consensus on treatment for such a kind of concomitant lesions. Until now, only a few studies and case reports have reported successful treatment of both tandem lesions in anterior circulation by a single-stage endovascular procedure (1–4). In addition, the effective treatment for such tandem lesions in posterior circulation is even rarely reported in the literature. Therefore, the efficacy and the safety of single-stage endovascular procedures under these conditions need to be further determined.

In the present study, we aimed to present a series of 10 patients with coexistence of severe cranial artery stenosis and ipsilateral distal tandem intracranial aneurysm. We also reviewed the literature to evaluate the safety and feasibility of a single-stage endovascular approach in treating tandem lesions.

## Materials and Methods

### Case Series

We retrospectively reviewed the available data in our database from January 2012 to June 2021 and retrieved 10 cases with the coexistence of severe cranial artery stenosis and ipsilateral distal tandem intracranial aneurysm. Clinical information, including age, sex, the onset of symptoms (artery stenosis or aneurysm), localization of artery stenosis and aneurysm, treatment modalities, and outcome of every single case, was analyzed and then compared with the published cases.

### Review of the Literature

We also performed a PubMed search to identify studies on the treatment of severe cranial artery stenosis associated with ipsilateral distal tandem intracranial aneurysm published between 1990 and 2021. Analysis was performed within studies of humans published in the English language. The studies were identified by using the keywords “(carotid OR vertebral OR subclavian artery stenosis) AND (aneurysm) AND (coincident OR coexist OR concomitant OR simultaneous OR ipsilateral).” Different forms of spelling (e.g., coincidental, coexistence, etc.) were considered.

**Figure 1:**
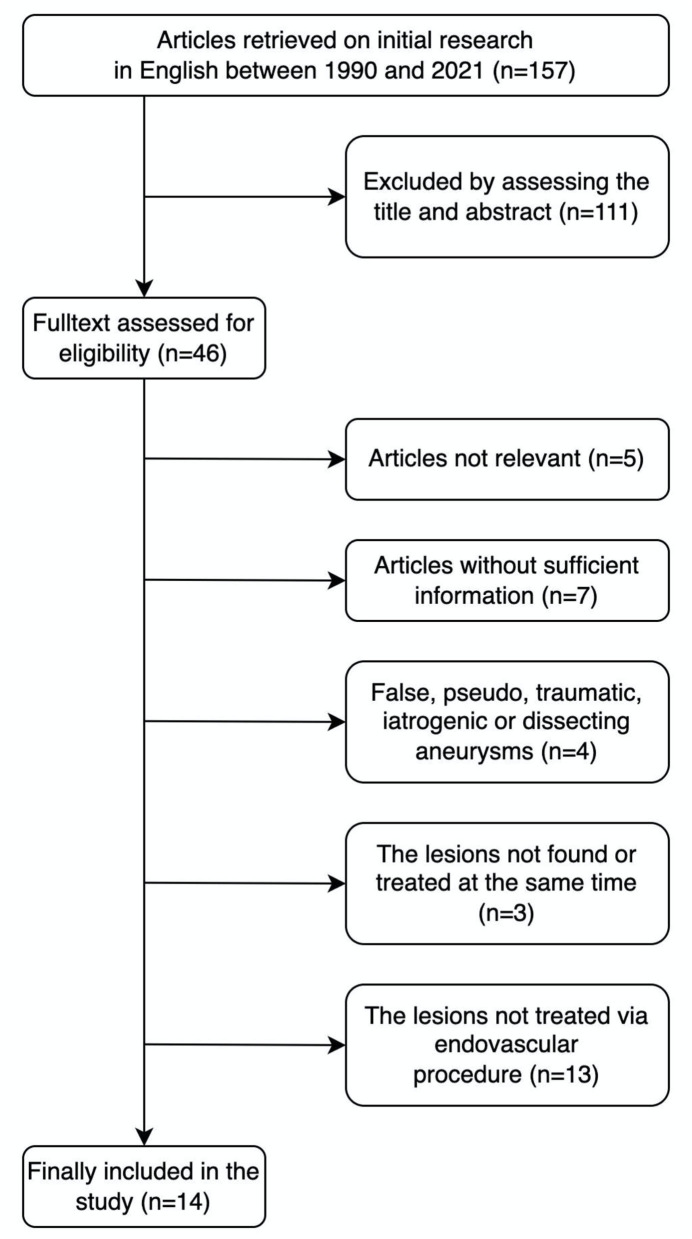
A flowchart of the literature-selection process.

Our initial search yielded 157 articles. In the investigated literature, only the studies providing enough information about the treatment procedure were taken for further analysis. Duplicate articles were removed. The studies were excluded according to the following exclusion criteria: (a) false, pseudo, traumatic, iatrogenic or dissecting aneurysm, (b) the coexistence of artery stenosis and intracranial aneurysm was not found or treated at the same time, and (c) the lesions were not treated *via* the endovascular procedure (Figure 1).

A total of 14 studies on 40 patients with the coexistence of severe cranial artery stenosis and ipsilateral distal tandem intracranial aneurysm were considered for analysis (1–14). All the studies were retrospective. Clinical information in these publications were also analyzed in the same way.

### Statistical Analysis

Statistical analysis was performed with SPSS Statistics (version 20.0, SPSS Inc., Chicago, IL, USA). Continuous variables were given as means and standard deviation. Categorical variables were summarized by numbers and percentages. The good prognosis was defined by a modified Rankin Scale (mRS) = 0 without complications. The Chi-square test was performed to test the differences of prognosis between single-stage and multiple-stage treatment. Significance of differences was defined as *p* < 0.05, two-tailed.

## Illustrative Case Reports

### Case 1

**Figure 2:**
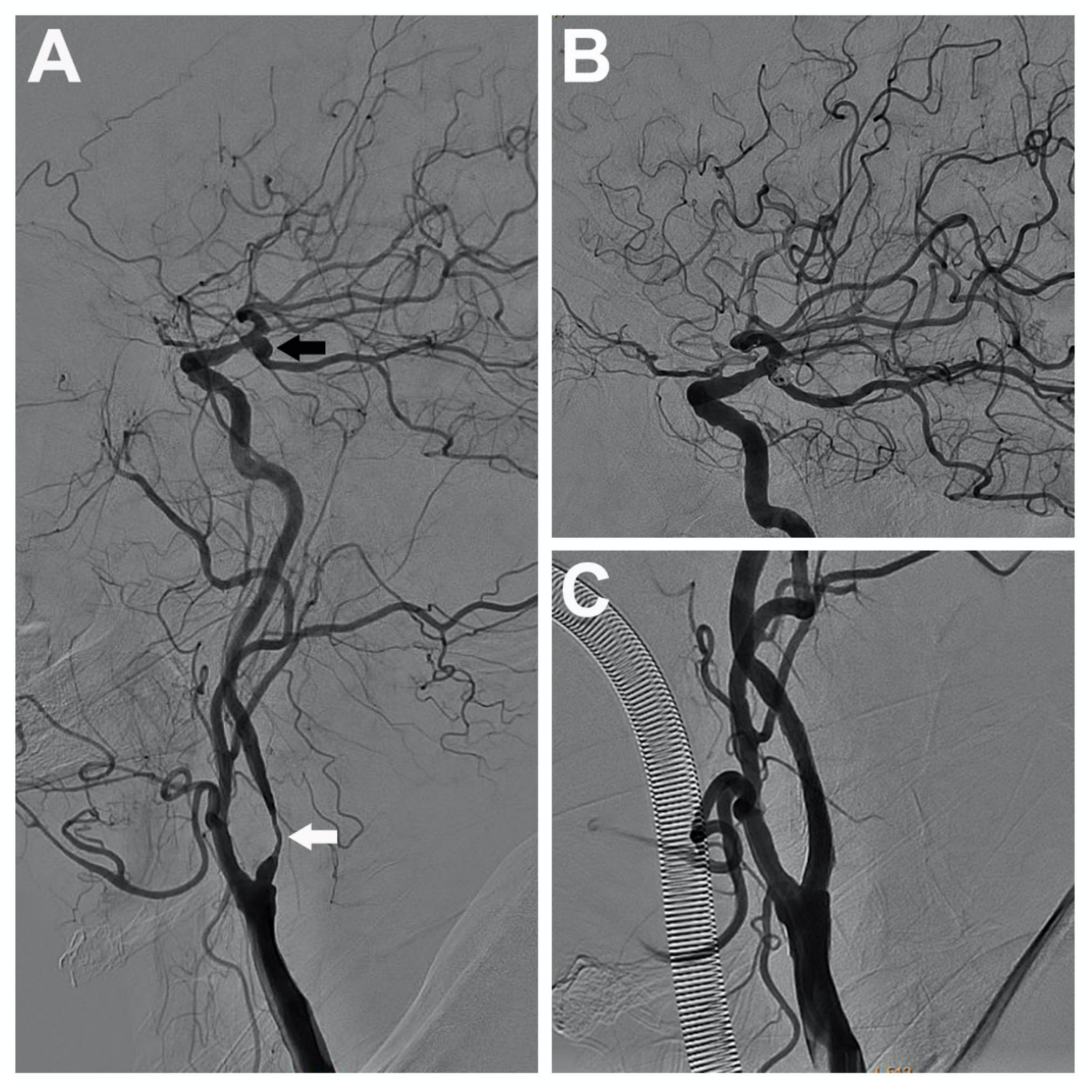
A 70-year-old man with severe stenosis in the left internal carotid artery (the white arrow) coexisted with an ipsilateral unruptured posterior communicating aneurysm (the black arrow), measuring 5 mm **(A)**. A single-stage endovascular procedure was implemented. After stent placement of the left internal carotid artery, stent-assisted embolization of the ipsilateral tandem aneurysm was applied. After single-stage treatment, left common carotid artery and internal carotid artery angiograms showed complete aneurysm exclusion **(B)** and satisfactory carotid revascularization **(C)**.

A 70-year-old man with a history of left-sided carotid transient ischemic attacks was found to have a 90% stenosis in the left internal carotid artery coexisted with an ipsilateral unruptured posterior communicating artery aneurysm, measuring 5 mm on angiography (Figure 2). A single-stage endovascular procedure was then planned. The patient was treated with aspirin and clopidogrel perioperatively, and the antiplatelet treatment was continued for 6 months post-operatively. During the procedure, the patient was anticoagulated using unfractionated heparin to achieve activated clotting time of 250–300 s. Also, the blood pressure was strictly monitored and treated. After initial carotid angioplasty and stenting, stent-assisted embolization of the posterior communicating aneurysm was applied successfully. Post-operatively, the patient recovered uneventfully without complication. At a 2-year follow-up, no re-stenosis, aneurysm expansion, or coil compaction was observed.

### Case 2

**Figure 3:**
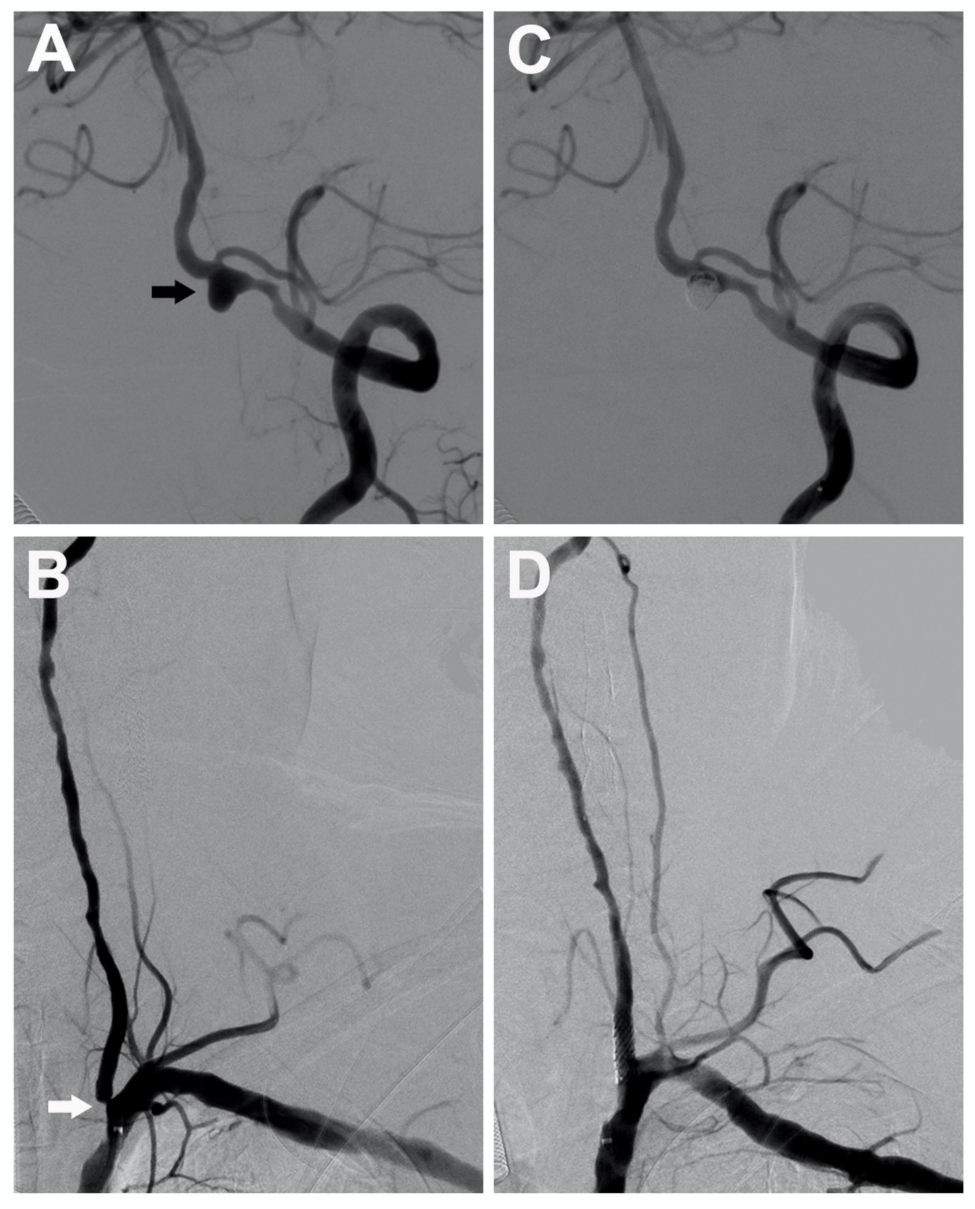
A 75-year-old man with severe stenosis in the left vertebral artery origin (the white arrow) coexisted with an ipsilateral unruptured vertebral aneurysm (black arrow), measuring 5.5 mm **(A,B)**. A single-stage endovascular procedure was implemented. After stent-assisted embolization of the ipsilateral tandem aneurysm, stent placement of left vertebral artery origin was applied. After single-stage treatment, the left subclavian artery angiogram showed complete aneurysm exclusion **(C)** and satisfactory vertebral revascularization **(D)**.

A 75-year-old man with episodes of recurrent drop attacks and orthostatic dizziness was found to have an 80% stenosis in the starting segment of the left vertebral artery coexisted with an ipsilateral unruptured vertebral aneurysm, measuring 5.5 mm on angiography (Figure 3). A single-stage endovascular procedure was recommended. The patient was treated with aspirin and clopidogrel perioperatively, and the antiplatelet treatment was continued for 6 months post-operatively. During the procedure, the patient was anticoagulated using unfractionated heparin to achieve activated clotting time of 250–300 s. Also, the blood pressure was strictly monitored and treated. At first, we dilated the proximal vertebral stenosis only enough to facilitate the microcatheter and microwire passing through the stenosis, but the stent implantation was not implemented at the time. After the stent-assisted embolization of the aneurysm, the vertebral stenosis was further dilated, and a stent was implanted subsequently. Post-operatively, the patient recovered well without complication. At the 6-month follow-up, no re-stenosis, aneurysm expansion, or coil compaction was found on angiography.

### Case 3

**Figure 4:**
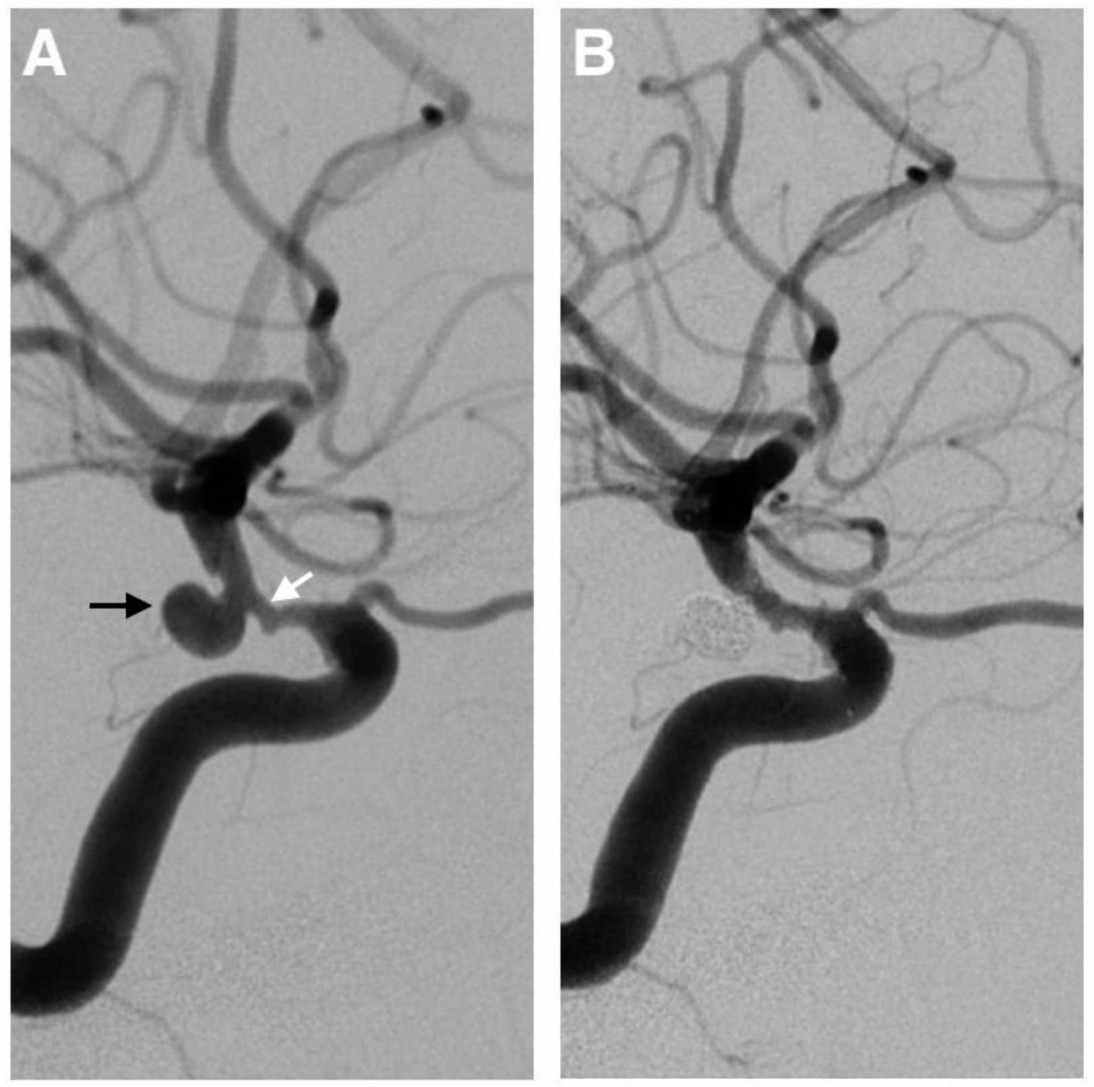
A 72-year-old man with a 6.4-mm posterior communicating aneurysm on the left internal carotid artery (the black arrow) accompanied by an 80% stenosis (the white arrow) proximal to the aneurysm **(A)**. A single-stage endovascular procedure was implemented. The stent-assisted embolization of the aneurysm was performed, and the stent was placed to cover both the neck of the aneurysm and the stenotic segment. After the operation, the left carotid artery angiogram showed complete aneurysm exclusion and satisfactory carotid revascularization **(B)**.

A 72-year-old man came with some recurrent involuntary twitches in the right arm. An MRI scan showed a subacute infarction in the left frontal-parietal lobe. On cerebral angiography, the patient was found to have a 6.4-mm posterior communicating aneurysm on the left internal carotid artery accompanied by an 80% stenosis proximal to the aneurysm (Figure 4). A single-stage endovascular procedure was recommended. The patient was treated with aspirin and clopidogrel perioperatively, and the antiplatelet treatment was continued for 6 months post-operatively. During the procedure, the patient was anticoagulated using unfractionated heparin to achieve activated clotting time of 250–300 s. Also, the blood pressure was strictly monitored and treated. We first dilated the proximal stenosis. Then we guided the microcatheter into the aneurysm and the stent to cover both the neck of the aneurysm and the stenotic segment. The procedure of stent-assisted embolization of the aneurysm was performed with the stent being released in the place as expected. Post-operatively, the patient recovered well without complication. At 1-year follow-up, no re-stenosis, aneurysm expansion, or coil compaction was found on angiography.

## Results

### Patients' Characteristics

An analysis of the available data showed that the coexistence of severe cranial artery stenosis and ipsilateral distal tandem intracranial aneurysm was found in 15 female and 24 male patients (one patient's gender is not available in the investigated literature), with a mean age of 65.5 years (range, 42–84 years). In the majority of the patients (*n* = 26/40, 65%), symptoms became apparent because of severe artery stenosis, and, in 4 cases (n = 4/40, 10%), symptoms were attributed to intracranial aneurysms.

The group of patients in our department consists of 6 women and 4 men. The mean age of the patients was 67.8 years (range, 57–75 years). In all 10 patients (*n* = 10/10), the symptoms were referred to severe artery stenosis (Tables 1, 2).

**Table 1:** Characteristics of our case series.

**Case**	**Age/sex**	**Symptoms by**	**Presentation**	**A location**	**S location**	**A size (mm)**	**S degree (%)**
1	70/F	S	Infarction	Lt clinoid	Lt ICA origin	5.2	85
2	61/M	S	TIAs	Rt clinoid	Rt ICA origin	4.5	90
3	65/F	S	TIAs	Rt cavernous	Rt ICA origin	6.8	90
4	72/M	S	Infarction	Lt PCom	Lt ICA C6	6.4	80
5	71/F	S	TIAs	Rt ophthalmic	Rt ICA origin	5.0	75
6	75/F	S	TIAs	Rt clinoid	Rt cavernous	4.8	75
7	70/M	S	TIAs	Lt PCom	Lt ICA origin	5.3	85
8	63/F	S	TIAs	Lt cavernous	Lt ICA origin	7.6	80
9	57/F	S	TIAs	Rt cavernous	Rt ICA C3	5.2	80
10	74/M	S	TIAs	Lt VA V4	Lt VA origin	5.5	85

*A, aneurysm; ICA, internal carotid artery; Lt, left; PCom, posterior communicating; Rt, right; S, stenosis; TIAs, transient ischemic attacks; VA, vertebral artery*.

**Table 2:** Patients' characteristics and outcomes.

**Parameters**	**Cases from literature** **(*n* = 40)**	**Our cases** **(*n* = 10)**	**Total** **(*n* = 50)**
Age, y	42–84	57–75	42–84
Sex, *n* (%)			
Men	24 (60.0)	4 (40.0)	28 (56.0)
Women	15 (37.5)	6 (60.0)	21 (42.0)
N/A	1 (2.5)	0 (0.0)	1 (2.0)
Symptoms by, *n* (%)			
Stenosis	26 (65.0)	10 (100.0)	36 (72.0)
Aneurysm	4 (10.0)	0 (0.0)	4 (8.0)
Occasionally	10 (25.0)	0 (0.0)	10 (20.0)
Location, *n* (%)			
Anterior circulation	37 (92.5)	9 (90.0)	46 (92.0)
Posterior circulation	3 (7.5)	1 (10.0)	4 (8.0)
Treatment procedure, *n* (%)			
Single-stage	35 (87.5)	10 (100.0)	45 (90.0)
Multiple-stage	5 (12.5)	0 (0.0)	5 (10.0)
Outcome, *n* (%)			
mRS <3	38 (95.0)	10 (100.0)	48 (96.0)
mRS ≥ 3	2 (5.0)	0 (0.0)	2 (4.0)

*N/A, not available; mRS, modified Rankin Scale*.

### Features of Intracranial Aneurysm and Artery Stenosis

In the reviewed literature, the size of aneurysm was >10 mm in 3 patients (*n* = 3/40, 7.5%), 5.1–10 mm in 23 patients (*n* = 23/40, 57.5%), and ≤ 5 mm in 14 patients (*n* = 14/40, 35%). The coexisted aneurysm mostly located in the anterior circulation (n = 37/40, 92.5%). Only 3 coexisted aneurysms (*n* = 3/40, 7.5%) were found in the posterior circulation. The carotid artery stenosis was detected in 37 patients (*n* = 37/40, 92.5%), while the vertebral artery or subclavian artery stenosis was found in 3 patients (*n* = 3/40, 7.5%).

In our group, the size of aneurysm was 5.1–10 mm in 7 patients (*n* = 7/10), and ≤ 5 mm in 3 (*n* = 3/10). None of the aneurysms were larger than 10 mm. The coexisted intracranial aneurysms were located in the anterior circulation in 9 patients (*n* = 9/10), and one aneurysm was found in the posterior circulation (*n* = 1/10). Severe carotid artery stenosis was demonstrated in 9 patients (*n* = 9/10), and severe vertebral artery stenosis was found in one patient (*n* = 1/10) (Table 2).

### Treatment

Our analysis of the available data showed that a single-stage endovascular procedure was applied in 35 patients (*n* = 35/40, 87.5%), while five patients (*n* = 5/40, 12.5%) received multiple-stage endovascular treatment. In the patients treated with the single-stage endovascular procedure, there were 31 patients (*n* = 31/35, 88.6%) who received proximal cranial artery stenting in the first place followed by the embolization of the aneurysm, while the other 4 patients (*n* = 4/35, 11.4%) were treated in a reversed manner. In the patients treated with a multiple-stage endovascular procedure, the proximal cranial artery stenting was performed as the first step in four patients (*n* = 4/5, 80%), while only one patient (*n* = 1/5, 20%) was treated first for the intracranial aneurysm.

In our case series, single-stage endovascular treatment was performed on all the patients (*n* = 10/10). In nine patients (*n* = 9/10) with tandem lesions in the anterior circulation, the proximal carotid artery stenosis was treated first (dilatation with a balloon and implantation of a stent), followed by the embolization of the distal intracranial aneurysm. In one patient (*n* = 1/10) with severe stenosis of the starting segment of the left vertebral artery accompanied with an ipsilateral V4 segment intracranial aneurysm, we first dilated the stenosis of the starting segment of the vertebral artery only enough to facilitate the microcatheter and the microwire passing through. After definite embolization of the intracranial aneurysm, the vertebral stenosis was further dilated, and the stent was implanted subsequently (Tables 3, 4).

**Table 3:** The treatment and the outcome of our case series.

**Case**	**Antiplatelet**	**Intra-op medication**	**Treatment**	**Complications**	**Outcome (mRS)**
1	Asp &Clo 3 days pre-op	Heparin	1st CAS, 2nd A embolization	None	0
2	Asp &Clo 3 days pre-op	Heparin	1st CAS, 2nd A embolization	None	0
3	Asp &ticagrelor 3 days pre-op	Heparin	1st CAS, 2nd A embolization	None	0
4	Asp &Clo 3 days pre-op	Heparin	1st CAS, 2nd A embolization	None	0
5	Cilostazol &Clo 3 days pre-op	Heparin	1st CAS, 2nd A embolization	None	0
6	Asp &Clo 3 days pre-op	Heparin	1st CAS, 2nd A embolization	None	0
7	Asp &Clo 3 days pre-op	Heparin	1st CAS, 2nd A embolization	None	0
8	Asp &Clo 3 days pre-op	Heparin	1st CAS, 2nd A embolization	None	0
9	Asp &Clo 3 days pre-op	Heparin	1st CAS, 2nd A embolization	None	0
10	Asp &Clo 3 days pre-op	Heparin	1stVA dialation, 2ndA embolization, 3rd VAS	None	0

*A, aneurysm; Asp, aspirin; CAS, carotid artery stenting; Clo, clopidogrel; mRS, modified Rankin Scale; op, operation; VAS, vertebral artery stenting*.

**Table 4:** Characteristics and outcomes of the single-stage procedure and the multiple-stage procedure.

**Parameters**	**Single-stage procedure** (*n* = 45)^*^	**Multiple-stage procedure** **(*n* = 5)**
Age, y	65.5 ± 9.6	65.2 ± 5.7
Sex, *n* (%)		
Men	27 (60.0)	1 (20.0)
Women	17 (37.8)	4 (80.0)
N/A	1 (2.2)	0 (0.0)
Symptoms by, n (%)		
Stenosis	32 (71.1)	4 (80.0)
Aneurysm	4 (8.9)	0 (0.0)
Occasionally	9 (20.0)	1 (20.0)
Location, *n* (%)		
Anterior circulation	41 (91.1)	5 (100.0)
Posterior circulation	4 (8.9)	0 (0.0)
Treatment procedure, *n* (%)		
Stenosis first	40 (88.9)	4 (80.0)
Aneurysm first	5 (11.1)	1 (20.0)
Outcome, *n* (%)		
mRS <3	43 (95.6)	5 (100.0)
mRS ≥ 3	2 (4.4)	0 (0.0)
Complications, n (%)	5 (11.1)	0 (0.0)

*N/A, not available; mRS, modified Rankin Scale*.

* *Including cases from reviewed literature and our case series*.

### Outcome

By analyzing the available data in the literature, we found that 95% of the patients (*n* = 38/40) achieved an excellent clinical outcome (mRS = 0). No death was observed. Rates of mRS = 0 outcomes were 100 and 94.3% for a multiple-stage procedure and a single-stage procedure, respectively. The main perioperative or post-operative complications were enlargement or rupture of the intracranial aneurysm (*n* = 2/40, 5%), re-stenosis after stenting (n = 1/40, 2.5%), and cerebral ischemia (*n* = 2/40, 5%).

In the present study, the mean clinical follow-up period was 23.6 months (range, 6–60 months). No perioperative or post-operative complications were observed. All the patients underwent angiographic follow-up at the 1st and 6th months post-operatively. No re-stenosis was observed in the patients with cranial artery stent. All the aneurysms were embolized completely, and no coil compaction was occurred.

Furthermore, the prognosis was compared between the patients with single-stage and with multiple-stage endovascular treatment. No differences were found though in the two groups (*p* = 1.000). Since all the patients treated with the multiple-stage endovascular procedure were found to have severe stenosis, we further compared the prognosis of severe stenosis between the two groups and found no differences (*p* = 1.000) (Table 4).

## Discussion

It is difficult to determine the true prevalence of the coexistence of severe cranial artery stenosis and ipsilateral distal tandem intracranial aneurysm. By reviewing the literature, we observed the fact that symptomatic presentation was more often caused by severe cranial artery stenosis in the majority of the patients, and coexisted ipsilateral distal tandem intracranial aneurysms were usually detected occasionally. But coexisted ipsilateral tandem intracranial aneurysm could still cause symptoms or even rupture (1, 4), although it was in the territory of the severe stenotic proximal cranial artery with theoretically decreased cerebral blood flow. Such tandem intracranial aneurysms are mostly located at the range of the circle of Willis. Therefore, it indicated that the redistribution of cerebral blood flow following unilateral proximal artery stenosis might affect the tandem intracranial aneurysm (especially at the range of the circle of Willis) distal to the ipsilateral stenosis through other vessels and the circle of Willis.

The coexistence of severe cranial artery stenosis and ipsilateral distal tandem intracranial aneurysm may result in a dilemma regarding the treatment. There is still a lack of consensus on whether to treat the occasionally detected lesion or not, and if it is needed to treat the occasionally detected lesion, what kind of procedure would be selected? According to the guidelines, all symptomatic unruptured aneurysms, incidental aneurysms larger than 5 mm in the patients younger than 60 years old, and incidental aneurysms >10 mm in all the healthy patients younger than 70 years old should be treated (15). Then, it raises the question of how should an incidental intracranial aneurysm <5 mm be managed in patients with ipsilateral extracranial artery stenosis. Theoretically, increased cerebral blood flow after revascularization of proximal extracranial artery stenosis may increase the risk of rupture of the intracranial aneurysm. Also, several studies reported that unruptured intracranial aneurysm <5 mm in diameter increased in size or ruptured after ipsilateral carotid endarterectomy (13, 16, 17). By further analysis of these studies, we found that all these aneurysms were at the range of the circle of Willis, and the subarachnoid hemorrhage from these aneurysms occurred within 30 days after the procedure of carotid revascularization. We, therefore, think that the incidental intracranial aneurysm <5 mm but within the range of the circle of Willis should be treated more actively.

After analyzing the available data from the literature and our own cases, we suggest that a single-stage endovascular procedure is a recommendable choice to treat both lesions. Compared with that of a multiple-stage procedure, the outcome of single-stage endovascular treatment showed no significant difference in neither post-operative complications nor prognosis. Moreover, we found that the single-stage endovascular treatment was effective for tandem lesions of the posterior circulation as well. A single-stage procedure could not only eliminate the need for an additional admission but also eliminate the further cost and discomfort of the patients (1).

In the single-stage endovascular procedure, the revascularization of the proximal cranial artery stenosis is usually applied at the first step, followed by the treatment of the ipsilateral distal tandem intracranial aneurysm. Initial revascularization of the stenotic artery is thought to have several advantages, including facilitating the subsequent procedure of the intracranial aneurysm coiling and reducing the thromboembolic risk related to catheter manipulation while passing through the stenosis (1, 2). In our case series, for patients with tandem lesions in the anterior circulation, the stenosis of carotid artery was firstly treated with balloon dilatation and stent implantation, then followed by the embolization of the distal intracranial aneurysm. However, for patients with posterior circulation tandem lesions, we chose a different strategy. Based on our experience, dilating the vertebral artery will increase cerebral blood perfusion but have few effects on blood pressure, which is different from dilating the carotid bifurcation. Considering that several studies have reported intracranial hemorrhage due to hyperperfusion following vertebral stenting (18, 19), a sudden increase in cerebral blood perfusion, which may cause the rupture of the tandem intracranial aneurysm in the posterior circulation, should be prevented. Moreover, the implantation of stent in the proximal vertebral artery prior to the embolization of distal aneurysm may make the guiding catheter, the microcatheter, and the microwire a little difficult to pass through the stent. Thus, we prefer to initially dilate the proximal vertebral stenosis just enough to facilitate the guiding catheter passing through the stenosis, while the stent was not implemented at that time. After definite embolization of the intracranial aneurysm, the vertebral stenosis would be further dilated and the stent would be implanted subsequently under stable control of blood pressure. Our experience can be a supplement to the literature as it proves the effectiveness and safety of the single-stage endovascular procedure for ipsilateral lesions in both anterior and posterior circulations.

The study has several limitations though. First, the present study was retrospective, contrasting with the well-designed, prospective, randomized comparative clinical trials. Methodological bias could influence the possible advantage of one treatment in comparison with the other. Second, although we combined our cases with the cases from the literature, the total number of cases and the available information were limited, which could influence the effectiveness of the statistical analysis. Furthermore, the functional outcome between a single-stage and multiple-stage is limited by the lack of sufficient data. However, the single-stage offers the advantages of 1 session and prevents the theoretical risk of aneurysm rupture after flow alteration. The advantage of single-stage endovascular treatment should be further confirmed and validated by future studies.

## Conclusion

The single-stage endovascular procedure is technically feasible and effective to treat the coexistence of severe cranial artery stenosis and ipsilateral distal tandem intracranial aneurysm. Initial revascularization of the proximal stenosis is recommendable in the patients with the tandem lesions in the anterior circulation; however, embolization of the intracranial aneurysm firstly seems to be preferable to treat the tandem lesions in the posterior circulation.

## Data Availability Statement

The original contributions presented in the study are included in the article/Supplementary Material, further inquiries can be directed to the corresponding author/s.

## Author Contributions

HN and ZZ analyzed the data and were the major contributors in writing the manuscript. JZ and HJ provided assistance for data acquisition and literature search. JH, DL, and LB carried out supervision and project administration. DL performed the manuscript review. All authors read and approved the final manuscript.

## Conflict of Interest

The authors declare that the research was conducted in the absence of any commercial or financial relationships that could be construed as a potential conflict of interest.

## Publisher's Note

All claims expressed in this article are solely those of the authors and do not necessarily represent those of their affiliated organizations, or those of the publisher, the editors and the reviewers. Any product that may be evaluated in this article, or claim that may be made by its manufacturer, is not guaranteed or endorsed by the publisher.
