# Supplementary material for: Single-Stage Endovascular Treatment of Severe Cranial Artery Stenosis Coexisted With Ipsilateral Distal Tandem Intracranial Aneurysm
Source: Front Neurol. 2022 May 18;13:865540. doi: 10.3389/fneur.2022.865540 (PMC9157431; doi:10.3389/fneur.2022.865540)
Supplement: Supplementary file 1 [file Table_1.DOCX]

Supplementary Table 1 Available data from patients with cranial artery stenosis coexisted with ipsilateral tandem intracranial aneurysm in the literature

| Study | Age/Sex | Symptoms by | Presentation | Location | | A size (mm) | S degree (%) | Stage | Treatment | Outcome (mRS) & Complications |
| --- | --- | --- | --- | --- | --- | --- | --- | --- | --- | --- |
|  |  |  |  | A | S |  |  |  |  |  |
| Pappada 1996 | 59/M | S | TIAs | Lt ICA bifurcation | Lt ICA origin | 5.0 | >90 | Multiple | 1^st^ CAS, 2^nd^ A embolization | 0 |
| Teitelbaum 1998 | 83/F | A | SAH | Rt MCA | Rt carotid bifurcation | 10.0 | 60 | Single | 1^st^ CAS, 2^nd^ A embolization | 4 / Rt frontal stroke |
| Navaneethan 2006 | 65/M | S | TIAs | Lt MCA | Lt ICA | 25.0 | 75 | Single | CAS & A embolization | 0 |
| Zappoli Thyrion 2007 | 63/F | / | Asymptom | Lt C6 segment | Lt ICA origin | 5.0 | >90 | Multiple | 1^st^ CAS, 2^nd^ A embolizaiton | 0 |
| Iwata 2008 | 69/F | S | TIAs | Lt ophthalmic | Lt ICA origin | 6.0 | 80 | Multiple | 1^st^ A embolization, 2^nd^ CAS | 0 |
| Gallego León 2009 | 45/F | S | Infarction | Lt ophthalmic | Lt ICA | 5.0 | 98 | Single | 1^st^ CAS, 2^nd^ A embolization | 0 |
|  | 51/M | S | TIAs | ACom | Rt ICA | 4.0 | 95 | Single | 1^st^ CAS, 2^nd^ A embolization | 0 |
|  | 76/F | S | Infarction | Lt PCom | Lt ICA origin | 7.0 | 90 | Single | 1^st^ CAS, 2^nd^ A embolization | 0 |
|  | 62/F | S | Headache | Rt ACho, Rt MCA | Rt ICA | 3.0/10.0 | 90 | Single | 1^st^ CAS, 2^nd^ A (ACho) embolization | 0 |
|  | 56/M | S | TIAs | ACom | Rt ICA origin | 6.0 | 80 | Single | 1^st^ CAS, 2^nd^ A embolization | 0 |
| Espinosa 2009 | 73/F | S | TIAs | Lt ophthalmic | Lt ICA origin | 14.0 | >90 | Multiple | 1^st^ CAS, 2^nd^ A embolization | 0 |
| Park 2013 | 67/M | S | TIAs | Rt ophthalmic | Rt ICA | 5.4 | 70 | Single | 1^st^ CAS, 2^nd^ A embolization | 0 / In-stent thrombosis |
|  | 42/F | S | TIAs | ACom | Rt ICA | 5.7 | 78 | Single | 1^st^ CAS, 2^nd^ A embolization | 0 |
|  | 69/M | S | TIAs | Lt PCom | Lt ICA | 4.0 | 73 | Single | 1^st^ CAS, 2^nd^ A embolization | 0 |
|  | 55/M | S | TIAs | Rt MCA | Rt ICA | 5.2 | 74 | Single | 1^st^ CAS, 2^nd^ A embolization | 0 |
|  | 69/M | S | TIAs | ACom | Lt ICA | 5.4 | 70 | Single | 1^st^ CAS, 2^nd^ A embolization | 0 |
|  | 69/M | S | TIAs | ACom | Lt ICA | 9.1 | 85 | Single | 1^st^ CAS, 2^nd^ A embolization | 0 |
|  | 65/M | S | TIAs | Lt ophthalmic | Lt ICA | 8.3 | 63 | Single | 1^st^ CAS, 2^nd^ A embolization | 0 |
|  | 65/M | S | TIAs | Lt PCom | Lt ICA | 6.2 | >90 | Single | 1^st^ CAS, 2^nd^ A embolization | 0 |
|  | 75/F | A | Oculomotor palsy | Rt PCom | Rt ICA | 7.7 | 50 | Single | 1^st^ CAS, 2^nd^ A embolization | 0 / Premature rupture of A |
|  | 65/M | / | Asymptom | ACom | Rt ICA | 4.4 | 80 | Single | 1^st^ CAS, 2^nd^ A embolization | 0 |
|  | 74/M | / | Asymptom | Rt A2-3 | Rt ICA | 5.7 | 62 | Single | 1^st^ CAS, 2^nd^ A embolization | 0 |
|  | 60/F | / | Asymptom | Lt ACho  Lt MCA | Lt ICA | 3.7/4.1 | 70 | Single | 1^st^ CAS, 2^nd^ A embolization | 0 |
|  | 66/M | / | Asymptom | Rt PCom | Rt ICA | 8.0 | 60 | Single | 1^st^ CAS, 2^nd^ A embolization | 0 / Recurrence of A |
|  | 70/M | / | Asymptom | ACom | Rt ICA | 5.0 | 60 | Single | 1^st^ CAS, 2^nd^ A embolization | 0 |
|  | 68/M | / | Asymptom | ACom  Lt MCA | Lt ICA | 4.2/3.4 | 52 | Single | 1^st^ CAS, 2^nd^ A embolization | 0 |
|  | 55/F | / | Asymptom | ACom  Rt A2-3 | Rt ICA | 4.1/6.3 | 50 | Single | 1^st^ CAS, 2^nd^ A embolization | 0 |
| Guo 2014 | 46/M | S | TIAs | Lt V4 | Lt adjacent V4 | 5.0 | >75 | Single | A embolization & S stenting | 0 |
|  | 57/M | A | SAH | Rt V4 | Rt adjacent V4 | 4.9 | >75 | Single | A embolization & S stenting | 0 |
| Kacar 2015 | 58/M | S | TIAs | ACom | Rt ICA origin | 6.0 | 80 | Single | 1^st^ CAS, 2^nd^ A embolization | 0 |
|  | 75/M | S | TIAs | Rt ACho | Rt ICA origin | 13.0 | 95 | Single | 1^st^ CAS, 2^nd^ A embolization | 0 |
|  | 73/M | S | TIAs | Rt PCom | Rt ICA origin | 9.5 | 76 | Single | 1^st^ CAS, 2^nd^ A embolization | 0 |
|  | 64/F | S | VBI | Proximal BA | Lt proximal subclavian artery | 7.0 | 90 | Single | 1^st^ S stenting, 2^nd^ A embolization | 0 |
|  | 53/M | S | TIAs | Lt paraclinoid | Lt ICA origin /Rt ICA origin | 6.0 | 70/95 | Single | 1^st^ CAS, 2^nd^ A embolization | 0 |
|  | 84/F | S | TIAs | Rt PCom | Rt ICA origin /Lt ICA origin | 5.0 | 70/80 | Single | 1^st^ CAS, 2^nd^ A embolization | 0 |
|  | 75/M | S | TIAs | Rt ophthalmic | Rt ICA origin | 8.0 | 79 | Single | 1^st^ CAS, 2^nd^ A embolization | 0 |
| Cvetic 2016 | 73/M | / | Asymptom | Rt supraclinoid | Rt ICA origin | 9.0 | 99 | Single | 1^st^ CAS, 2^nd^ A embolization | 0 |
| Wajima 2019 | 62/F | S | Rt hand numbness | Lt PCom | Lt ICA origin | 9.0 | 95 | Multiple | 1^st^ CAS, 2^nd^ A embolization | 0 |
| Campos 2018 | N/A | / | Asymptom | Rt ACho, Rt PCom | Rt ICA origin | 4.2/5.0 | 62 | Single | 1^st^ CAS, 2^nd^ A embolization | 0 |
| Guo 2019 | 74/F | A | Lt eyelip ptosis, SAH | Lt PCom, Rt PCom | Lt ICA origin | 8.0/3.8 | ≥90 | Single | CAS & A (Lt) embolization | 3-4 / Lt temporal infarction |

A, aneurysm; ACho, anterior choroidal; ACom, anterior communicating; BA, basilar artery; CAS, carotid artery stenting; F, female; ICA, internal carotid artery; Lt, left; M, male; MCA, middle cerebral artery; mRS, modified Rankin Scale; O, occasionally; PCom, posterior communicating; Rt, right; S, stenosis; SAH, subarachnoid hemorrhage; TIAs, transient ischemic attacks; VBI, vertebrobasilar insufficiency
